# Intrahepatic Cholestasis of Pregnancy with Severe Elevation of Bile Acids in the Setting of Acute Hepatitis C Infection

**DOI:** 10.1155/2016/4963283

**Published:** 2016-11-06

**Authors:** Megan L. Lawlor, Agatha S. Critchfield

**Affiliations:** Department of Obstetrics and Gynecology, Division of Maternal Fetal Medicine, University of Kentucky College of Medicine, Lexington, KY, USA

## Abstract

Intrahepatic cholestasis of pregnancy (ICP) is a complication of pregnancy resulting in elevation of serum bile acid levels. ICP is often associated with underlying liver disease, including hepatitis C. Bile acids in relationship to the acute infection of hepatitis C virus have not yet been delineated in the literature. A 26-year-old gravida 4 para 2103 with dichorionic, diamniotic twin gestation and history of intravenous drug abuse developed ICP in the setting of acute hepatitis C infection. In addition to clinical symptoms of pruritus and right upper quadrant pain, she developed severe elevation in bile acids, 239 micromol/L, and transaminitis aspartate aminotransferase 1033 U/L, and alanine aminotransferase 448 U/L. She received ursodeoxycholic acid and antenatal testing was performed. Patient delivered vaginally at 33-week gestation following preterm rupture of membranes. Neonates were admitted to NICU and had uncomplicated neonatal courses. In the setting of ICP with significant transaminitis and severe elevation of bile acids, consideration of acute viral hepatitis is important, especially considering the worsening opioid epidemic and concurrent increase in intravenous drug use in the United States. Further study is needed regarding the acute form of HCV infection and its effect on ICP and associated bile acids.

## 1. Introduction

Intrahepatic cholestasis of pregnancy (ICP) is a complication of pregnancy resulting in diffuse pruritus, elevation of serum bile acid levels, and potential serum transaminitis. ICP is the most common liver disease diagnosed during pregnancy, and typically, symptoms manifest in the 2nd or 3rd trimester of pregnancy. Elevations in bile acids have been shown to be associated with adverse pregnancy outcomes, most notably meconium staining of amniotic fluid, fetal asphyxial events, spontaneous preterm delivery, iatrogenic preterm delivery, and sudden intrauterine fetal demise [1]. A review of the literature demonstrates that ICP may be associated with underlying liver disease, including hepatitis C virus (HCV) infection. However, much of the literature does not specifically investigate the acute versus chronic forms of HCV infection in relationship to ICP. According to the guidelines by the American Association for the Study of Liver Diseases and the Infectious Diseases Society of America, anti-HCV is the first-line diagnostic test for patients with elevated liver enzymes or clinical signs of liver disease [2]. A positive HCV antibody result does not distinguish between acute and chronic infection of HCV or from a previous infection that has been cleared spontaneously [2]. Acute hepatitis C infection can be diagnosed by the presence of HCV RNA by polymerase chain reaction (PCR) and the absence of HCV antibodies. HCV RNA is detectable by PCR within days to eight weeks following exposure, while most patients seroconvert to HCV antibody positive between two and six months after exposure [3, 4]. The presence of serum HCV antibodies may be delayed in those who are immunocompromised or pregnant [2]. Of note, approximately 20–50% of patients will spontaneously clear the HCV infection, with the majority occurring within 6 months of exposure [5]. The purpose of this report is to add to our understanding of the clinical course of concurrent ICP and the acute form of HCV infection during pregnancy.

## 2. Case

A 26-year-old gravida 4 para 2103 with a dichorionic, diamniotic pregnancy developed intrahepatic cholestasis in the setting of acute HCV infection. Her obstetrical history was notable for her three previous pregnancies complicated by preeclampsia. Her past medical history included opioid abuse for 7 years including daily intravenous drug use, as well as tobacco abuse, asthma, and depression. She denied any personal or family history of liver disease.

At 21 weeks of gestation, the patient was admitted for voluntary detoxification from intravenous opiate abuse. During this admission, the patient reported significant right back pain and right upper quadrant abdominal pain. The patient was found to have transaminitis of unclear etiology, with respective levels of AST and ALT of 886 U/L and 405 U/L. Evaluation at that time included a negative hepatitis panel and unremarkable right upper quadrant ultrasound. The negative hepatitis panel demonstrated absence of serum antibodies for hepatitis A, hepatitis B, and hepatitis C and absence of hepatitis B surface antigen. During her admission, the patient was transitioned from detoxification to buprenorphine for opioid maintenance therapy. Her abdominal pain resolved spontaneously and the patient was discharged home. At 22 weeks of gestation, the patient presented to a routine prenatal visit with a complaint of diffuse itching. Ursodeoxycholic acid 300 mg orally three times daily was initiated empirically for a clinical presentation concerning ICP. A repeat hepatitis panel was again negative; however HCV RNA PCR was positive, with viral load of approximately 3 million IU/mL. Initial level and also highest recorded level of serum bile acids was 239 micromol/L; AST and ALT reached peak levels of 1033 U/L and 448 U/L, respectively. The patient was continued on ursodeoxycholic acid and received standard antenatal testing. Fetal status remained reassuring throughout her pregnancy, as evidenced by nonstress tests and serial ultrasounds. Growth percentiles were 46% and 49% at 27 weeks of gestation, for twin A and twin B, respectively. At 33 weeks of gestation, growth percentiles were 31% and 35% for twin A and twin B, respectively. Weight discordance between twins remained at an estimated 3 percent throughout the duration of the pregnancy. Bile acid levels declined to 12 micromol/L and AST and ALT improved to 21 U/L and 10 U/L, respectively, at 28 weeks of gestation.

At 33 weeks and 6 days of gestation, the patient presented with preterm premature rupture of membranes and subsequent preterm delivery of twins. She had uncomplicated spontaneous vaginal deliveries: twin A was a 1790 g female neonate with Apgar scores of 7 and 9, and twin B was a 1800 g male neonate with Apgar scores of 6 and 7. Thin meconium was noted at the time of delivery of twin A. Neonates were admitted to the NICU for preterm status and had uncomplicated neonatal courses. Placental pathology revealed a dichorionic, diamniotic twin placenta with significant findings including dystrophic calcification and increased syncytial knotting. Specifically, syncytial knots are a marker of placental maturity and are also strongly associated with preeclampsia [6].

Maternal postpartum course was complicated by gestational hypertension, which required oral nifedipine extended release 30 mg twice daily for treatment of elevated blood pressures. The patient otherwise recovered well in the immediate postpartum period, during which pruritus resolved spontaneously. Approximately 9 months postpartum, HCV RNA PCR was negative, indicating spontaneous clearance of the HCV infection.

## 3. Discussion

A comprehensive review of the literature was performed using PubMed with the search terms “intrahepatic cholestasis,” “obstetric cholestasis,” “pregnancy,” and “hepatitis C.” Our case represents an isolated report of intrahepatic cholestasis of pregnancy with concurrent acute HCV infection, notably with a severe elevation of serum bile acid levels.

A strong association exists between ICP and underlying hepatobiliary dysfunction. In terms of HCV infection and ICP, a reciprocal relationship may exist. A cohort study by Marschall et al. suggests that the high prevalence of HCV infection with ICP may be due to enhanced susceptibility to HCV in pregnancies affected by ICP, and vice versa [7]. Patients with chronic hepatitis tend to have clinical presentation of ICP at earlier gestational ages as compared to those patients without HCV [8]. In addition, pregnant women with concurrent HCV infection have higher hepatitis C viral load values than patients with HCV infection without ICP. Several researchers have advocated for routine testing of hepatitis in pregnancies complicated by ICP [7, 8]. Furthermore, our case highlights the potential need for reflexive HCV RNA PCR testing to rule out acute viral infection if initial hepatitis antibody panels are negative.

The extent to which ICP or hepatitis affects bile acid levels remains uncertain. Proposed mechanisms of the pathophysiology of ICP include genetic predisposition with subsequent variants of hepatobiliary transport proteins, impaired turnover of reproductive hormones, and environmental factors [7]. Furthermore, bile acids in relationship to acute HCV infection have not yet been elucidated in the literature. HCV infection may downregulate expression of the ATP-binding cassette transporter MRP2 found in the liver, resulting in a relative failure in the transport of various toxins. This failure combined with high circulating serum estrogen levels during pregnancy may increase the risk of ICP and may enhance symptoms of cholestatic pruritus [9].

An increased incidence of ICP has been demonstrated among twin pregnancies [10]. In our case, the severity of bile acid elevation may be confounded by multiple factors including the patient's multifetal gestation, history of preeclampsia, the uncertain timeline of her acquisition of the hepatitis C virus, and the acute nature of the HCV infection. Specifically, Wikström Shemer et al. have demonstrated an increased incidence of preeclampsia among pregnancies affected by ICP, with adjusted odds ratio 2.62 [11]. Additionally, a recent retrospective cohort study of 78 patients with ICP has revealed that severe ICP (total bile acid level greater than 40 micromol/L) is noted as a significant risk factor for preeclampsia in both singleton and twin pregnancies [12]. Interestingly, patients with bile acid levels less than 20 did not develop preeclampsia in this study [12]. As seen in our case, normalization of bile acid levels and liver function tests typically occurs within one to three weeks of treatment with ursodeoxycholic acid [12]. Despite normalization of these laboratory values, the development of preeclampsia remains a subsequent event later in pregnancy, perhaps suggesting that the initial elevation in bile acids may act to promote the development of hypertensive disorders of pregnancy [12]. Further studies are necessary to delineate the causative factors contributing to bile acids reaching severe levels in ICP.

Many studies have examined bile acid levels in association with adverse pregnancy outcomes. A positive linear correlation exists between bile acid levels and adverse fetal outcomes including meconium staining of amniotic fluid, fetal asphyxial events, spontaneous preterm delivery, iatrogenic preterm delivery, and sudden intrauterine fetal demise [1, 13]. Several studies have delineated levels of bile acids in terms of severity and subsequent fetal complication rates. A prospective cohort study by Glantz et al. suggested an additional one to two percent increased risk of adverse events for each additional micromol/L of bile acid level above 40 micromol/L [1]. In 2015, Brouwers et al. conducted a retrospective cohort study that demonstrated an increased risk of adverse fetal complications with bile acids greater than 100 micromol/L [14]. Additionally, this study noted a positive correlation among maternal and fetal bile acid levels and revealed that, for each 10 micromol/L increase of bile acids, there is an increased risk of perinatal death (*P* = 0.039), with an odds ratio of 1.26 when adjusted for gestational age, maternal age, and birthweight [14]. This is equivalent to a 10% risk of stillbirth if bile acids are greater than 100 micromol/L [14]. Comparably, a 2015 retrospective cohort study by Kawakita et al. reaffirmed that bile acid levels greater than 40 micromol/L were associated with increased risk of meconium-stained amniotic fluid (OR 3.55, CI 1.45–8.68), as well as the association between bile acids greater than 100 *μ*mol/L and an increased risk of stillbirth [13].

While the relationship of bile acid levels and adverse pregnancy outcomes is clear, the timing for optimal delivery for patients with ICP has not yet been formally established. Multiple studies have demonstrated benefit for delivery at 37 weeks of gestation if bile acids are greater than 40 micromol/L [1, 14]. Brouwers et al. proposed late preterm delivery between 34 and 37 weeks of gestation for pregnancies complicated by bile acids greater than 100 micromol/L in the setting of ICP [14]. Important to consider is the temporality of bile acid elevation during pregnancy. As emphasized in our case, does a rapid onset of bile acid elevation affect rates of fetal complications?

Furthermore, patients with ICP are at risk of subsequent development of cirrhosis and other liver, biliary, and pancreatic diseases [15]. Specifically considering concomitant acute HCV infection, postpartum follow-up and further management are likely to be beneficial for these patients.

## 4. Conclusions

In the setting of ICP with significant transaminitis and severe levels of bile acids, consideration of acute viral hepatitis is important. In particular with the increasing opioid epidemic and concurrent increase in intravenous drug use in the United States, the prevalence of acute HCV infection may be increasing among pregnant women. Reflexive hepatitis C RNA PCR testing to rule out acute viral infection if initial hepatitis antibody panels are negative may be warranted in patients with ICP and concurrent severe elevation in bile acid levels. Diagnosis of acute HCV infection may help to distinguish a subset of patients with ICP and severe levels of bile acids; this may result in alteration of guidelines for optimal delivery planning. The effect of acute HCV infection on the setting of ICP in relation to severity of bile acid elevation and associated adverse pregnancy complications deserves further study.
